# Sensory Quality Evaluation of Korla Pear from Different Orchards and Analysis of Their Primary and Volatile Metabolites

**DOI:** 10.3390/molecules25235567

**Published:** 2020-11-27

**Authors:** Yuan Liu, Simin Xiang, Haipeng Zhang, Hongyan Zhang, Cuiyun Wu, Zhanghu Tang, Jiangbo Wang, Juan Xu

**Affiliations:** 1 Key Laboratory of Horticultural Plant Biology (Ministry of Education), College of Horticulture and Forestry Sciences, Huazhong Agricultural University, Wuhan 430070, China; luiyuer@webmail.hzau.edu.cn (Y.L.); ansimiy@163.com (S.X.); haipengzhang@webmail.hzau.edu.cn (H.Z.); zhanghy@mail.hzau.edu.cn (H.Z.); 2 Research Group on Sensory Evaluation and Consumer Preference of Horticultural Products, College of Horticulture and Forestry Sciences, Huazhong Agricultural University, Wuhan 430070, China; 3 The National and Local Joint Engineering Laboratory of High Efficiency and Superior-Quality Cultivation and Fruit Deep Processing Technology of Characteristic Fruit Trees in South Xinjiang, College of Plant Science, Tarim University, Alar 843300, China; wcyby@163.com; 4 Xinjiang Production and Construction Corps Key Laboratory of Biological Resources Protection and Utilization in Tarim Basin, Alar 843300, China; 5 National Fruits Germplasm Resources Garden of Xinjiang Academy of Agricultural Sciences in Luntai, Luntai 841600, China; ligu@webmail.hzau.edu.cn

**Keywords:** metabolites, Korla pear fruits, sensory evaluation, fuzzy logic model

## Abstract

Metabolites play vital roles in shaping the quality of fresh fruit. In this study, Korla pear fruit harvested from twelve orchards in South Xinjiang, China, were ranked in sensory quality by fuzzy logic sensory evaluation for two consecutive seasons. Then, gas chromatography-mass spectrometry (GC-MS) was applied to determine the primary metabolites and volatile compounds. Sensory evaluation results showed that the panelists were more concerned about ‘mouth feel’ and ‘aroma’ than about ‘fruit size’, ‘fruit shape’ and ‘peel color’. In total, 20 primary metabolites and 100 volatiles were detected in the pear fruit. Hexanal, (*E*)-2-hexenal, nonanal, d-limonene, (*Z*)-3-hexen-1-yl acetate and hexyl acetate were identified as the major volatile compounds. Correlation analysis revealed that l-(+)-tartaric acid, hexanoic acid, trans-limonene oxide and 2,2,4-trimethyl-1,3-pentanediol diisobutyrate were negatively correlated with sensory scores. Furthermore, OPLS-DA results indicated that the fruit from three orchards with lower ranks in quality could be distinguished from other samples based on the contents of l-(+)-tartaric acid and other eight metabolites, which were all associated with ‘mouth feel’ and ‘aroma’. This study reveals the metabolites that might be closely associated with the sensory quality attributes of Korla pear, which may provide some clues for promoting the fruit quality in actual production.

## 1. Introduction

Korla pear (*Pyrus sinkiangensis* Yu) is a distinctive and economic cultivar widely planted in Xinjiang, China, and is popular among domestic consumers due to its unique flavor, refreshing taste and crispy texture. Moreover, a large amount of Korla pear fruit are exported to foreign countries every year. However, the quality of Korla pear is hardly unified due to the vast planting territory in Xinjiang region with varied climate and soil conditions.

Aroma and taste are important indicators of fruit quality, and are closely related to fruit metabolites due to the complex interactions between metabolites and human senses. Metabolites such as soluble sugars, organic acids and volatiles play important roles in fruit sensory quality. Although there has been extensive research on the metabolites in different pear cultivars [1,2,3,4,5,6,7], few studies were focused on the fruit sensory quality and relevant metabolites of Korla pear [8,9,10,11]. In Korla pear, aldehydes and esters such as hexanal and hexyl acetate are dominant volatile compounds [8,12,13]; while fructose and malic acid are the major soluble sugar and organic acid, respectively [3,5]. Compared with other pear cultivars, Korla pear has a higher sugar/acid ratio that contributes to its excellent taste [3].

Sensory evaluation plays a vital role in the assessment of food preference. Combination of chemical and statistical analysis with sensory evaluation can well demonstrate the association between the sense and compounds in food. Since information acquired from human senses cannot enable precise quantitative assessment, fuzzy logic has been employed for more reasonable ranking of food samples [14,15,16]. It has become a commonly used method for the sensory evaluation of foods such as beverages and tea products [17,18,19]. Nevertheless, it has only been applied to the sensory evaluation of a limited number of fresh fruit varieties [20,21,22], and is more often used to explore the correlations between sensory evaluation results and food chemical compounds [23,24,25,26]. In this study, we attempted to firstly evaluate the sensory quality attributes of Korla pear from 12 orchards, and then determined the primary and volatile metabolites. Finally, the pear fruit samples were ranked according to the sensory scores to determine whether the sensory quality attributes are related to the production areas and some key metabolites. The findings may help the regionalized planting of Korla pear in Xinjiang on the basis of fruit quality and sensory quality attributes, and provide theoretical support to promote and stabilize the fruit quality in actual production.

## 2. Results

### 2.1. Sensory Evaluation of Korla Pear

In the two seasons, the sensory scores of all samples were given by the panelists and presented in the Supplementary Material (Table S1). Triplets were obtained for the ranking of various sensory quality attributes (Table S2). The triplets for the overall quality scores of the samples were then generated in Table S3 based on the data in Tables S1 and S2. The values of overall membership function of sensory scores for all pear samples are presented in the Supplementary Material (Table S4), which were then used together with standard fuzzy scale to calculate the similarity values. The samples were graded on the standard fuzzy scale based on the maximum similarity values. Finally, pear fruit from different orchards within a same grade were ranked from 1 to 12 based on the similarity values (Table 1).

In 2018, S3 in the Akesu district and S11 in the Kuerle district ranked the top, while S5 in the Kuerle district ranked the last. In 2019, S11 in the Kuerle district and S12 in the Akesu district ranked the top, and S7 in the Akesu district was the last.

Sensory quality attributes were ranked based on the triplets by the same methods. The corresponding similarity values were compared (Table S5). ‘Mouth feel’ and ‘aroma’ ranked the top two in both 2018 and 2019, suggesting that flavor has great contribution to the preference of consumers for Korla pear.

### 2.2. Primary Metabolites in Korla Pear

Primary metabolites in the pulp of Korla pear harvested in 2018 were analyzed by using GC-MS (Table 2). As a result, seven amino acids, four organic acids, two fatty acids and seven sugars were detected (Table S6). In addition to the high content of malic acid and fructose, glucitol was also detected at a high level of 12.17–24.34 mg/g. Shikimic acid was undetectable. l-(+)-Tartaric acid was detected at a level of 2.36–7.79 μg/g, but it was rarely counted in most studies due to its low concentration. Galactose was detected at a level of 0.14–0.30 mg/g, while it was not considered in most studies owing to its low sweetness value [5,6]. The sugar/acid ratio of Korla pear ranged from 42.06 to 74.03, which was higher than that of many other pear varieties such as Dangshan, Yali and Nanguo pear cultivars [2,3,5], and thus might greatly contribute to its excellent quality [3].

### 2.3. Volatiles in Korla Pear

The volatiles were profiled in the peels of Korla pear harvested in 2018 from 12 orchards (Table S7). Although more than 200 volatile compounds had been reported in Korla pear [8,9,10,12,13,27], there exist varies between different Korla pear samples. A total of 100 volatile compounds were detected in this article, including nine aldehydes, 20 esters, 12 alcohols, 19 terpenes and 40 other volatiles such as alkanes. Aldehydes were the most abundant compounds among the volatiles (Table 3). A wide variety of esters were detected despite of their low contents. For example, hexyl acetate was detected at 82.43–818.52 μg/kg. Many of them such as hexyl acetate usually contribute to the impression of ‘fruity’ in sensory evaluation [12]. A number of terpenes which were usually detected at high concentrations in citrus essential oil were also detected in Korla pear. The concentrations of d-limonene and α-farnesene were 34.16–937.00 μg/kg and 0.00–277.18 μg/kg. In general, hexanal, (*E*)-2-hexenal, nonanal, d-limonene, (*Z*)-3-hexen-1-yl acetate and hexyl acetate were abundant. For pear fruit harvested from different orchards, the volatile profiles showed great variations. On average, the number of compounds detected in the pear fruit from all 12 orchards was 69. However, only 34 compounds were commonly detected in all samples, whereas seven were unique in pear fruit from certain orchards (Table S8). For example, tetradecanoic acid was only detected in S12 with a concentration of 1225.67 ± 825.43 μg/kg.

### 2.4. OPLS-DA Analysis on Korla Pear Fruit from 12 Orchards

**Figure 1:**
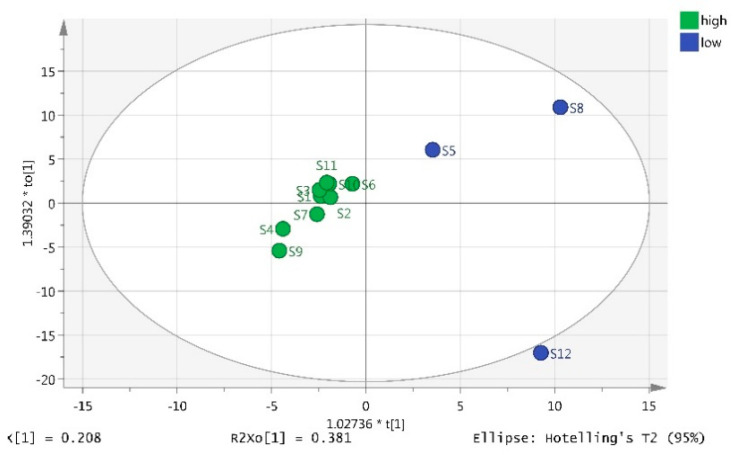
Score plot of OPLS-DA models with the statistical parameters (R2X = 0.208, R2Y = 1.000, Q2 = 0.321) for the classification of Korla pear fruit. High: pear fruit ranked 1–9 in sensory score; Low: pear fruit ranked 10–12 in sensory score.

In order to discriminate Korla pear fruit with different sensory qualities, the GC-MS data including the concentrations of primary metabolites and volatiles were analyzed by using OPLS-DA. As shown in Figure 1, the pear fruit could be classified into two groups, with one corresponding to high sensory quality (sensory ranks: 1–9) and the other corresponding to low sensory quality (sensory ranks: 10–12). As a result, nine metabolites with VIP values higher than 1.5, including l-(+)-tartaric acid, malic acid, d-(−)-fructofuranose, α-bulnesene, fructose, psicopyranose, l-proline, glucose and l-α-terpineol, were obtained and listed in the Supplementary Material (Table S9).

### 2.5. Correlation Analysis between Sensory Scores and Metabolites

**Figure 2:**
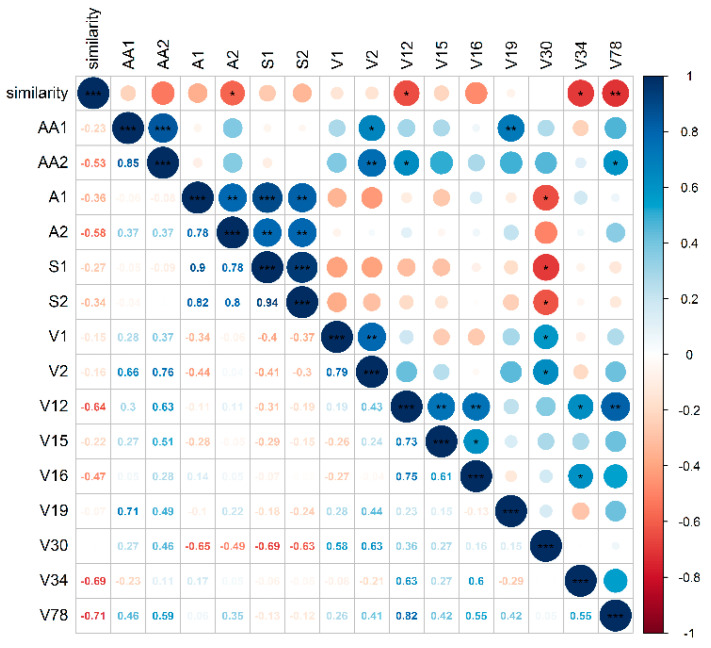
Correlation analysis of sensory scores and metabolites. AA1 and AA2 were amino acids, A1 and A2 were organic acids, S1 and S2 were soluble sugars listed in Table S6; V1, V2, V12, V15, V16, V19, V30, V34, V78 were volatiles presented in Table S7. * Significantly correlated at the 0.05 level. ** Significantly correlated at the 0.01 level. *** Significantly correlated at the 0.001 level.

The similarity values and metabolite concentrations of Korla pear fruit from 12 orchards were subjected to Pearson correlation analysis. Figure 2 presents part of the correlation coefficients. None of the primary metabolites except for l-(+)-tartaric acid was found to be correlated with sensory scores. For volatiles, hexanoic acid, trans-limonene oxide and 2,2,4-trimethyl-1,3-pentanediol diisobutyrate showed negative correlations with sensory scores.

## 3. Discussion

### 3.1. Important Sensory Quality Attributes Defined in the Study

The Kuerle district is thought to be a traditionally superior production area of Korla pear, and the pear fruit are sold out quickly in each season with better prices. However, pear fruit from this area did not exhibit its superiority in this study. Moreover, the ranks of sensory quality for pear fruit from the same orchard were not consistent in two consecutive seasons. For example, the rank of S5 changed from the twelfth to the fourth, while that of S6 shifted from the third to the eleventh, which may be ascribed to the variations in the fruiting of trees due to poor orchard management. Additionally, there were only subtle differences among pear fruit from 12 orchards as most samples were classified into F4/Good. Although the deficiency in the precision of evaluation by panelists could be somewhat compensated by using fuzzy logic, differences between pear samples had not been amplified as expected, possibly because of their identical genotype.

Currently, the price of Korla pear in the market mainly depends on the fruit size and shape, which is also the case for many other fruits, mainly because other attributes such as ‘aroma’ can hardly be quantified by a unified standard. However, ‘mouth feel’ and ‘aroma’ were more important than other three attributes in this study, probably because all the panelists chosen for the test were young college students with basic knowledge of horticultural products. Their choices may partly represent the preference of young people. It could be speculated that the flavor and mouth feel may become important sensory quality attributes of Korla pear in the future, and the findings in this study may promote the improvement of market standards for the production of Korla pear.

### 3.2. Metabolites Detected in Korla Pear

The amino acid content determined in this study was generally lower than that previously described [3]. The lack of acid hydrolysis process may lead to insufficient release of amino acids. Compared with the results of a previous study [27], the acids were reduced while the sugars showed no significant change in the period from commercial maturity (160 DAFB, days after full bloom) to physiological maturity (180 DAFB) in the pulp of Korla pear. As a result, there was a further increase in sugar/acid ratio at physiological maturity of 180 DAFB. The high sugar/acid ratio may be the major reason for ‘mouth feel’ to rank the first in the five quality attributes.

The volatiles previously reported to be associated with the flavor of Korla pear, such as hexanal, (*E*)-2-hexenal, hexyl acetate, and α-farnesene, were also detected in this study [8,12,13]. The number of volatiles detected in the fruit from different orchards ranged from 57 (in S5) to 84 (in S8), implying that the Korla pear fruit from different orchards had unique volatile profiles. As for different developmental stages, the total volatile content rose by nearly 10 folds from commercial maturity to physiological maturity [27]. Since all volatiles of Korla pear showed sharp increases in this period, it could be speculated that the period from 160 DAFB to 180 DAFB is a crucial period for the formation of its flavor. For Korla pear, the primary metabolite profiles were relatively similar among all samples, while volatile profiles were diverse, indicating that volatiles are more susceptible to environment factors.

### 3.3. Association between Metabolites and the Sensory Quality of Korla Pear

Four metabolites, including l-(+)-tartaric acid, hexanoic acid, trans-limonene oxide and 2,2,4-trimethyl-1,3-pentanediol diisobutyrate (TXIB), were found to be negatively correlated with sensory scores. TXIB was known as a kind of plasticizer and could be an ambient environmental pollutant which contribute to indoor odor and irritation [28], it’s sensory perception fitted well with the negative correlation. As TXIB was not a natural material, it was very possible to be a pollutant from human activities as the local farmers often spray something to help remove the calyx of Korla pear and shaping pear beautiful. However, there may be some more potential connections between metabolites and sensory quality or among various metabolites. As metabolites in food generally form a comprehensive flavor in various proportions, a single compound has limited impact on fruit quality. Other indicators such as the ratio between some key flavor metabolites can be taken into account in future research.

## 4. Materials and Methods

### 4.1. Plant Materials

**Figure 3:**
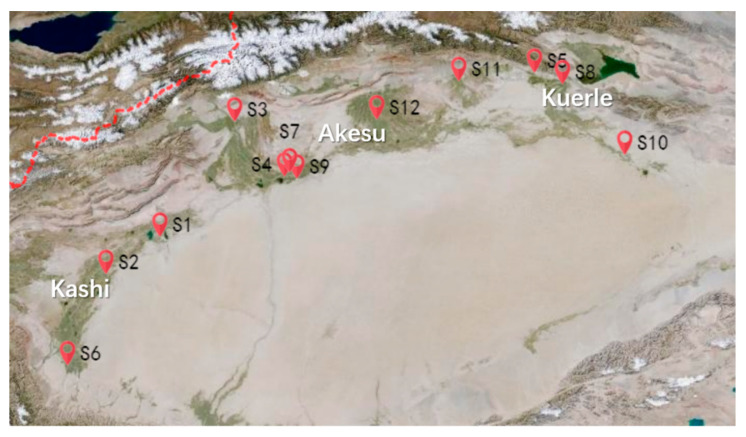
Geographic coordinates of the 12 pear orhchards in Xinjiang.

Fruit of Korla pear grown in the Kashi, Akesu and Kuerle districts in Circum-Tarim Basin, South Xinjiang of China were harvested from 12 different orchards (Figure 3) at two consecutive seasons in 2018 and 2019, respectively. All samples with representative size and shape of each orchard were harvested at commercial maturity on around 160 DAFB. For each orchard, thirty fruit were used for sensory evaluation in 2018 and 2019, respectively, and fifteen were used for the analysis of primary metabolites and volatiles in 2018.

### 4.2. Chemicals and Reagents

Methanol of HPLC grade was purchased from Fisher Scientific (Fair lawn, NJ, USA). Standard of *n*-paraffins mixture (C7-C40) were purchased from ANPEL Laboratory Technologies (Shanghai) Inc. (Shanghai, China). Sodium chloride was obtained from Sinopharm Chemical Reagent Co., Ltd. (Shanghai, China). Methyl nonanoate, Ribitol, *N*-methyl-*N*-(trimethylsilyl) trifluoroacetamide (MSTFA) and methoxamine hydrochloride were purchased from Sigma Aldrich (Saint Louis, MO, USA). All the chemicals and reagents were of analytical grade.

### 4.3. Sensory Evaluation of Korla Pear Fruit

Korla pear fruit were evaluated using the fuzzy logic model described in previous studies [17,18,19] in the Sensory Evaluation Laboratory of Key Laboratory of Horticultural Plant Biology (Ministry of Education), Huazhong Agricultural University. Sixty healthy panelists (at the age of 20–30 with equal numbers of male and female) were selected from untrained students in the College of Horticulture and Forestry Science, Huazhong Agricultural University, who were representatives of those educated young consumers with certain horticultural knowledge. The quality attributes selected for sensory evaluation included ‘peel color’, ‘fruit shape’, ‘fruit size’, ‘aroma’ and ‘mouth feel’. The above attributes were graded as ‘Poor,’ ‘Fair,’ ‘medium,’ ‘Good’ or ‘Excellent.’ The panelists were required to judge the importance of each attribute of the samples at five scales: ‘NI’ for not important, ‘SI’ for somewhat important, ‘IM’ for important, ‘HI’ for highly important and ‘EI’ for extremely important. Ten panelists were randomly selected to participate in each round of testing. In each evaluation of one sample, five fruit were presented to each panelist and then the panelist gave his evaluation results about the five sensory attributes, respectively. The panelists were asked to clean their mouth with water and have a 3 min-rest after tasting each sample in order to eliminate sensory fatigue. Totally, 20 datasets were collected for each sample.

**Figure 4:**
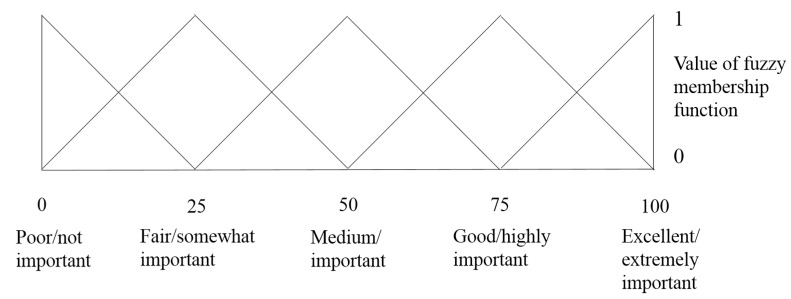
Triangular membership function distribution pattern of 5-point scale [17].

The major steps involved in the fuzzy modeling of sensory evaluation were: (1) calculation of the overall sensory scores in the form of triplets; (2) assessment of membership function on the standard fuzzy scale; (3) estimation of the overall membership function on the standard fuzzy scale; (4) calculation of the similarity values and ranking of the fruit samples from the 12 different orchards. The 5-point sensory scales were composed of ‘Poor’ (0 0 25), ‘Fair’ (25 25 25), ‘Medium’ (50 25 25), ‘Good’ (75 25 25) and ‘Excellent’ (100 25 0) (Figure 4). The first number of the triplets indicates the coordinate of the abscissa where the value of the membership function is 1, while the second and the third numbers represent the distance of the first number to the left and right, respectively, where the value of the membership function is 0.

#### 4.3.1. Calculation of the Overall Quality Scores

For each sample, the triplets corresponding to sensory quality attributes were calculated by the following equation:

SC = (n1 (0 0 25) + n2 (25 25 25) + n3 (50 25 25) + n4 (75 25 25) + n5 (100 25 0))/(n1 + n2 + n3 + n4 + n5)

where SC is the triplet for ‘peel color’ attribute, ‘n1′ to ‘n5′ are the numbers of panelists who chose a certain grade for each sensory quality attribute of each sample. The first number was normalized to obtain the triplets for relative weightage.

After that, triplets for all sensory quality attributes were generated, and the triplets for the overall quality scores could be calculated by the following equation:

SO = SC × QCr + SS × QSr + SD × QDr + SA × QAr + ST × QTr

where SO is the overall quality triplet, ‘C’ is for ‘peel color’, ‘S’ for ‘fruit shape’, ‘D’ for ‘fruit size’, ‘A’ for ‘aroma’, ‘T’ for ‘mouth feel’. SC, SS, SD, SA and ST are the triplets for quality attributes, respectively. ‘QCr’, ‘QSr’, ‘QDr’, ‘QAr’ and ‘QTr’ represent the triplets for relative weightage of their corresponding quality attributes, respectively. The multiplication of two triplets was conducted as

(a b c) × (d e f) = (a × d a × e + d × b a × f + d × c).

#### 4.3.2. Calculation of Membership Function on the Standard Fuzzy Scale

The standard fuzzy scale of (F1, F2, F3, F4, F5, F6) follows the triangular distribution pattern, where the maximum value of membership function is 1, and the values were defined by a set of 10 numbers.

F1 = (1 0.5 0 0 0 0 0 0 0 0), F2 = (0.5 1 1 0.5 0 0 0 0 0 0), F3 = (0 0 0.5 1 1 0.5 0 0 0 0) F4 = (0 0 0 0 0.5 1 1 0.5 0 0), F5 = (0 0 0 0 0 0 0.5 1 1 0.5) and F6 = (0 0 0 0 0 0 0 0 0.5 1)

For each sample with a triplet, its corresponding overall membership function value was calculated with the formulas below:

If (a − b) < x < a, Bx = (x − (a − b))/b; if a < x <(a + c), Bx = ((a + c) − x)/c; if x = a, Bx = 1; if x < (a − b) or x > (a + c), Bx = 0.

For each sample and its triplets, the values of the membership function Bx at 0, 10, 20, 30, 40, 50, 60, 70, 80, 90 and 100 could be obtained through the above formula. The membership function value of samples on the standard fuzzy scale for matrix B1 to B12 would be given a set of 10 numbers, which were the maximum values of Bx at 10 intervals of 0 < x <10 to 90 < x < 100, respectively.

#### 4.3.3. Calculation of Similarity Values and Ranking of the Korla Pear Fruit from Different Orchards

The similarity values of pear fruit from different orchards were obtained with the following equation:

Sm = (F × B’)/(Max(F × F’ and B × B’))

where Sm is the similarity value of the samples, and F’ and B’ are the transpose of matrix F and B, respectively.

Six similarity values would be obtained for each sample, and then applied to the ranking of the pear fruit from different orchards.

### 4.4. Analysis of Primary Metabolites by Using GC–MS

The seeds, stones and peels were first removed, and then the fruit flesh was cubed. Primary metabolites in the cubes of Korla pear fruit were detected by GC-MS as described in previous studies [29,30] with minor modifications. The relative concentration of compounds was calculated by comparison with the internal standard. The cubes were quickly ground into powder with liquid nitrogen. Then, 0.30 g powder was thoroughly mixed with 3 mL methanol containing 0.02 mg/mL ribitol as the internal standard. After shaking and ultrasonic treatment for 30 min, the mixture was put into a thermostated water bath at 70 °C for 15 min and centrifuged at 5000 *g* for 15 min. Then, 100 μL supernatant were collected and vacuum-concentrated. Metabolites were trimethylsilylation (TMS) derivatized before GC-MS analysis. Samples were dissolved in 80 μL methoxamine hydrochloride (20 mg/mL in pyridine), incubated for 90 min at 37 °C, and then reacted with 80 μL of MSTFA for 30 min at 37 °C. After that, 1 μL of each sample was injected into the gas chromatography (Thermo Fisher Scientific, Waltham, MA, USA) onto a fused-silica capillary column (30 m × 0.25 mm i.d., 0.25 μm DB-5MS stationary phase). The temperature program of GC was as follows: 100 °C for 1 min, heated to 184 °C at 3 °C /min, heated to 190 °C at 0.5 °C /min and kept for 1 min, heated to 280 °C at 15 °C/min and kept for 5 min, with the pulsed split injector temperature held at 230 °C as the split ratio was 10, and the carrier gas was set at a flow rate of 1.2 mL/min. The conditions for mass spectrometer were: electron ionization source, electron energy of 70 eV; ionization temperature 260 °C, transfer line temperature 280 °C and scanning range of 40 to 650 amu.

The compounds detected by using GC-MS was identified by comparing their mass spectra with the NIST MS Search 2.3.

Semiquantitative determinations were conducted using ribitol as an internal standard. The contents of volatiles were calculated from the GC TIC peak areas related to the GC TIC peak area of the internal standard.

### 4.5. Volatile Determination by Headspace Solid Phase Microextraction-GC-MS

Since more than 90% of total volatiles were found in the peel, the volatiles in the peel of Korla pear were determined using a previously described method [27]. The relative concentration of compounds was calculated by comparison with the internal standard. The peel tissue was quickly ground into powder with liquid nitrogen. Then, 2.50 g powder was placed in 15-mL headspace vials together with 5 mL 30% NaCl solution containing 0.1 μL/100 mL methyl nonanoate as the internal standard. The vials were then placed on the platform for automatic sample injection. The sample in the vial was incubated at 45 °C for 15 min, and the Divinylbenzene/Carboxen/Polydimethylsiloxane fiber (50/30 μm, DVB/CAR on PDMS, 2 cm) was conditioned at 250 °C for 5 min for each sample before testing. Then the volatiles in the headspace of the vials were collected for 15 min by using the fiber. Subsequently, the fiber was removed from the vial and immediately inserted into the GC injection port to desorb the volatiles at 250 °C for 5 min. The analysis was performed with gas chromatography (Thermo Fisher Scientific, Waltham, MA, USA) equipped with a TRACE TR-5 MS capillary column (30 m × 0.25 mm × 0.25 μm, Thermo Scientific, Bellefonte, PA, USA). Helium was used as the carrier gas at a constant flow rate of 1 mL/min. The temperature program of GC was as follows: 40 °C for 3 min, heated to 160 °C at 3 °C/min and kept for 1 min, heated to 200 °C at 5 °C/min and kept for 1 min, heated to 240 °C at 8 °C/min e and kept for 1 min, with the pulsed splitless injector temperature held at 250 °C. The conditions for mass spectrometer were: electron ionization source, electron energy of 70 eV; ionization temperature 230 °C, transfer line temperature 230 °C and scanning range of 30 to 550 amu.

The compounds detected by using GC-MS was identified by comparing their mass spectra with the NIST MS Search 2.3. RIs was calculated basing on the retention time of C7–C40 alkane series under the same chromatographic conditions.

Semiquantitative determinations were conducted using methyl nonanoate as an internal standard. The contents of volatiles were calculated from the GC TIC peak areas related to the GC TIC peak area of the internal standard.

### 4.6. Statistical Analysis

The results were presented as means of three biological replicates ± standard deviation (SD). SIMCA 14.1, RStudio and Excel 2016 were used for all statistical analyses. Supervised orthogonal partial least-squares discriminate analysis (OPLS-DA) was applied to discriminate Korla pear fruit from different orchards with different sensory scores by using SIMCA 14.1. The corresponding variable importance in projection (VIP) value was calculated in the OPLS-DA model, which represented the differences of the variables. Compounds that played important roles in the grouping of fruit were picked out when the VIP value was higher than 1.5. Pearson correlation analysis was conducted with similarity values and metabolite concentrations using RStudio.

## 5. Conclusions

In the present study, the sensory quality of Korla pear fruit from 12 orchards in Circum-Tarium Basin in two consecutive seasons was ranked by using the fuzzy logic model. The ranks of sensory quality attributes indicated that ‘mouth feel’ and ‘aroma’ have great contribution to the fruit quality of Korla pear. Hexanal, (*E*)-2-hexenal, nonanal, d-limonene, (*Z*)-3-hexen-1-yl acetate and hexyl acetate were identified as the major volatiles in Korla pear. Notably, l-(+)-tartaric acid, hexanoic acid, trans-limonene oxide and 2,2,4-trimethyl-1,3-pentanediol diisobutyrate were found to be negatively correlated with the sensory similarity values.

## Figures and Tables

**Table 1:** Similarity values for pear samples.

Sample	F1/Not Satisfactory	F2/Fair	F3/Satisfactory	F4/Good	F5/Very Good	F6/Excellent	**Rank**
2018							
S1	0.014	0.171	0.465	**0.683**	0.544	0.169	9
S2	0.024	0.226	0.534	**0.687**	0.452	0.119	7
S3	0.025	0.237	0.564	**0.705**	0.430	0.096	1
S4	0.016	0.186	0.488	**0.690**	0.513	0.151	5
S5	0.008	0.136	0.409	**0.659**	0.597	0.199	12
S6	0.021	0.212	0.525	**0.694**	0.463	0.122	3
S7	0.018	0.193	0.492	**0.688**	0.507	0.147	6
S8	0.048	0.310	0.652	**0.675**	0.317	0.037	11
S9	0.026	0.238	0.550	**0.685**	0.441	0.114	8
S10	0.020	0.207	0.517	**0.691**	0.469	0.127	4
S11	0.014	0.180	0.494	**0.704**	0.512	0.143	2
S12	0.010	0.151	0.437	**0.675**	0.575	0.185	10
2019							
S1	0.024	0.254	0.630	**0.721**	0.373	0.057	5
S2	0.005	0.139	0.462	**0.710**	0.559	0.167	6
S3	0.024	0.244	0.594	**0.709**	0.411	0.087	7
S4	0.030	0.296	0.693	**0.698**	0.279	0.023	9
S5	0.022	0.241	0.610	**0.722**	0.399	0.074	4
S6	0.033	0.307	**0.683**	0.663	0.302	0.090	11
S7	0.038	0.311	**0.682**	0.675	0.28	0.026	12
S8	0.039	0.318	**0.686**	0.666	0.279	0.027	10
S9	0.019	0.221	0.578	**0.726**	0.430	0.09	3
S10	0.026	0.264	0.635	**0.708**	0.365	0.058	8
S11	0.007	0.162	0.518	**0.741**	0.486	0.112	1
S12	0.009	0.165	0.508	**0.733**	0.502	0.125	2

Note: bold numbers are the maximum similarity values of pear samples on the standard fuzzy scale.

**Table 2:** Primary metabolites detected in the pulp of Korla pear fruit by using GC-MS.

Compounds	RT	Concentration (μg/g FW)			
		Maximum	Sample	Minimum	Sample
**Amino acid**					
l-Valine	6.11	5.36 ± 0.29	S12	0.30 ± 0.25	S6
l-Isoleucine	8.12	1.01 ± 0.07	S12	-	S6
l-Proline	9.21	37.26 ± 0.70	S12	11.93 ± 0.98	S6
Serine	10.18	4.75 ± 0.21	S12	0.05 ± 0.07	S5
l-Threonine	11.00	2.81 ± 0.03	S12	0.02 ± 0.00	S3
l-Aspartic acid	15.67	52.88 ± 5.89	S12	1.32 ± 0.70	S5
l-Glutamic acid	19.24	4.73 ± 0.10	S12	0.09 ± 0.05	S6
**Organic acids**					
Malic acid	14.61	1809.13 ± 420.52	S8	600.20 ± 25.76	S9
l-(+)-Tartaric acid	18.51	7.79 ± 1.81	S8	2.36 ± 0.24	S2
Citric acid	26.21	31.40 ± 2.27	S11	12.35 ± 1.10	S9
Quininic acid	27.46	212.43 ± 162.28	S10	105.39 ± 20.03	S11
**Fatty acids**					
Palmitic Acid	34.41	14.20 ± 8.54	S11	3.64 ± 2.93	S6
Stearic acid	44.07	15.05 ± 9.92	S11	1.93 ± 1.71	S6
**Sugars**					
d-(−)-Fructofuranose	25.77	18492.86 ± 4309.41	S8	11285.72 ± 359.93	S4
Fructose	26.07	20999.04 ± 4646.51	S8	12012.71 ± 22.17	S4
Psicopyranose	27.11	11453.29 ± 2590.96	S8	6820.89 ± 394.51	S4
Glucose	28.81	13910.25 ± 3120.71	S8	8145.27 ± 138.45	S2
*d*-Galactose	29.31	303.88 ± 47.85	S8	139.70 ± 96.73	S11
d-Glucitol	30.26	24335.64 ± 5912.74	S8	12171.03 ± 8458.33	S11
Sucrose	48.42	2968.10 ± 246.14	S6	1900.73 ± 168.91	S12
Total Amino acid		108.82 ± 5.90	S12	14.65 ± 2.93	S6
Total acid		1099.01 ± 41.92	S10	806.63 ± 60.89	S9
Total sugar		91705.26 ± 20996.43	S8	56878.51 ± 2251.72	S4
Sugar/Acid		74.03	S9	42.06	S11

**Table 3:** Volatile compounds detected in Korla pear from 12 orchards.

Compounds	Retention Index (RI)	Concentration Range (μg/kg FW)	Average Concentration (μg/kg FW)/Percentage of Total Volatiles (%)
Aldehydes		10222.35–75589.04	26133.94/85.84
Hexanal	800	3472.75–35617.72	12954.25/42.55
(*E*)-2-Hexenal	854	2782.32–39086.40	12666.51/41.60
Octanal	1003	0.00–318.75	39.06/0.13
Nonanal	1104	211.58–798.32	416.57/1.37
Decanal	1206	7.91–144.89	28.74/0.09
2,6,10-trimethyl-9-Undecenal	1416	7.74–66.02	17.15/0.06
Tetradecanal	1613	0.16–41.36	6.50/0.02
1-Pentadecanal	1715	0.00–23.41	2.82/0.01
Hexadecanal	1817	0.00–24.99	2.35/0.01
**Esters**		**361.82–3206.45**	**1314.47/4.32**
Methyl hexanoate	925	0.00–11.27	1.62/0.01
Acrylic acid isoamyl ester	940	3.61–24.71	10.80/0.04
Ethyl hexanoate	1000	0.00–5.12	0.85/-
(*Z*)-3-Hexen-1-yl acetate	1005	152.86–2501.62	780.49/2.56
hexyl acetate	1011	82.43–818.52	345.54/1.13
(2*Z*)-2-Hexen-1-yl acetate	1016	0.00–275.32	72.59/0.24
Heptyl acetate	1113	0.00–22.30	8.38/0.03
Hexyl butyrate	1192	0.00–54.56	17.26/0.06
(*E*)-Butanoic acid-2-hexenyl ester	1195	1.16–31.02	9.32/0.03
Ethyl octoate	1196	0.00–0.54	0.07/-
Octyl acetate	1210	0.00–4.61	1.39/-
Acetic acid-2-phenylethyl ester	1258	0.00–22.06	5.85/0.02
n-Butyric acid 2-ethylhexyl ester	1317	0.00–21.71	7.95/0.03
Lavandulyl propionate	1375	0.00–14.75	1.74/0.01
Hexanoic acid hexyl ester	1384	0.00–36.33	9.35/0.03
(*E*)-Hexanoic acid-2-hexenyl ester	1391	0.00–5.13	1.47/-
Formic acid undecyl ester	1441	12.32–61.90	25.53/0.08
Benzoic acid hexyl ester	1580	0.00–13.77	1.60/0.01
2,2,4-Trimethyl-1,3-pentanediol diisobutyrate	1588	3.05–33.60	12.06/0.04
Propanoic acid 2-methyl-decyl ester	1590	0.00–2.92	0.63/-
**Alcohols**		**46.52–1214.99**	**315.82/1.04**
1-Hexanol	868	0.00–632.22	166.87/0.55
(*E*)-2-Octen-1-ol	1067	0.00–2.60	0.55/-
1-Octanol	1071	12.73–97.24	31.76/0.10
(*Z*)-3-Nonen-1-ol	1156	0.00–5.42	0.62/-
1-Nonanol	1173	13.23–147.34	45.37/0.15
1-methyl-4-(1-methylethyl)-Cyclohexanol	1178	0.00–35.01	2.92/0.01
9-Decen-1-ol	1262	0.00–25.53	4.39/0.01
1-Decanol	1273	0.00–38.34	10.15/0.03
1-Dodecanol	1473	7.35–52.17	16.38/0.05
1-Tetradecanol	1676	7.66–144.20	29.42/0.10
1-Hexadecanol	1880	0.00–54.61	8.18/0.03
1-Octadecanol	2082	0.00–17.92	2.12/0.01
**Terpenes**		**74.90–1220.67**	**482.98/1.59**
α-Thujene	929	1.93–15.79	5.98/0.02
d-Limonene		34.16–937.00	357.05/1.17
γ-Terpinene	1060	0.00–13.83	5.24/0.02
Linalool	1099	2.03–9.05	4.75/0.02
Isophorone	1124	0.00–67.36	6.49/0.02
trans-Limonene oxide	1138	0.00–7.14	0.79/-
l-α-Terpineol	1190	0.00–18.46	4.62/0.02
Geraniol	1255	0.00–4.36	0.96/-
cis-Geranylacetone	1435	0.00–65.67	17.84/0.06
cis-β-Farnesene	1444	0.00–0.15	0.01/-
Humulene	1454	0.00–31.44	9.76/0.03
(*E*)-2-Dodecenal	1468	0.00–8.26	1.02/-
(*Z*,*E*)-α-Farnesene	1491	0.00–2.96	0.25/-
α-Farnesene	1508	0.00–277.18	54.17/0.18
β-Curcumene	1514	0.00–3.80	0.97/-
d-Nerolidol	1544	0.00–29.25	4.73/0.02
Nerolidol	1554	0.00–14.44	3.04/0.01
Viridiflorol	1591	0.00–23.10	3.79/0.01
α-Bulnesene		0.00–6.57	1.51/-
**Others**		**502.16–15082.46**	**2197.67/7.22**
1,2-dimethyl-Benzene	887	0.00–108.07	31.80/0.10
methoxy-phenyl-Oxime		3.12–374.30	120.12/0.39
(*E*,*E*)-2,4-Hexadienal	911	0.00–1045.21	206.89/0.68
(*E*)-4-Oxohex-2-enal	958	55.12–1815.23	309.67/1.02
Phenol	980	0.00–0.41	0.07/-
Hexanoic acid	990	0.00–202.81	56.68/0.19
*p*-Cymene	1025	0.00–60.38	11.66/0.04
Benzeneacetaldehyde	1045	2.11–22.32	7.34/0.02
(*Z*)-1-ethoxy-4-methyl-2-Pentene		3.52–1941.80	354.24/1.16
1-methyl-4-(1-methylethylidene)-Cyclohexene	1088	0.00–2.22	0.23/-
2-ethenyl-1,4-dimethyl-Benzene	1090	0.00–0.37	0.03/-
2-Nonanone	1092	0.00–156.99	31.20/0.10
Undecane	1100	0.00–8.23	3.08/0.01
1,2,4,5-tetramethyl-Benzene	1116	0.00–35.93	12.78/0.04
Benzoic acid	1170	0.00–103.50	12.65/0.04
Naphthalene	1182	2.04–26.36	7.28/0.02
Dodecane	1200	9.03–77.73	34.46/0.11
(*Z*)-3,7-dimethyl-2,6-Octadienal	1240	0.00–4.56	2.05/0.01
Nonanoic acid	1273	0.00–26.95	4.73/0.02
2,6,11-trimethyl-Dodecane	1275	0.00–36.70	10.34/0.03
Tridecane	1300	7.44–58.40	18.34/0.06
2,3,5,8-tetramethyl-Decane	1318	0.00–27.36	7.97/0.03
Tetradecane	1400	18.28–91.39	35.06/0.12
Pentadecane	1500	0.00–231.63	60.09/0.20
2,6,10-trimethyl-Tetradecane	1539	0.00–19.23	6.37/0.02
5-methyl-Pentadecane	1547	2.21–45.84	7.54/0.02
5,8-Diethyldodecane	1572	3.79–34.53	9.61/0.03
Hexadecane	1600	5.84–64.97	15.45/0.05
6,9-Heptadecadiene	1667	0.00–2.30	0.56/-
1-Hydroxycyclohexyl phenyl ketone	1687	9.28–199.96	39.17/0.13
Tetradecanoic acid	1768	0.00–1225.67	102.14/0.34
2,6,11,15-tetramethyl-Hexadecane	1792	0.19–33.98	5.64/0.02
Pentadecanoic acid	1867	0.00–1175.82	98.01/0.32
Nonadecane	1900	3.85–94.82	18.62/0.06
9-Hexadecenoic acid	1942	0.00–1610.17	135.11/0.44
*n*-Hexadecanoic acid	1968	0.00–3839.76	319.98/1.05
Eicosane	2000	0.00–52.30	6.24/0.02
Heptadecanoic acid	2071	0.00–129.25	10.77/0.04
Heneicosane	2100	8.04–295.40	47.09/0.15
Oleic Acid	2141	0.00–404.28	33.69/0.11
**Total**		**13098.35–79188.69**	**30444.88/100.00**

Note: ‘-’ in the line of the percentage means that the percentage of compounds was lower than 0.005%; bold numbers were the total contents of different kinds of substances; the retention index was acquired on the semi-standard non-polar.

## Supplementary Materials

The following are available online. Table S1: Sensory scores for pear samples and the corresponding triplets; Table S2: Preferences given by panelists and corresponding triplets for relative weightage; Table S3: Overall triplets of pear samples; Table S4: Values of overall membership function of pear samples; Table S5: Similarity values of quality attributes for Korla pear; Table S6: Primary metabolites detected in the pulp of Korla pear by using GC-MS; Table S7: Volatiles detected in the peel of Korla pear by using HS-SPME-GC-MS; Table S8: Seven volatile compounds uniquely detected in specific orchards in 2018; Table S9: List of potential chemical markers.
